# Tau Phosphorylation and μ-Calpain Activation Mediate the Dexamethasone-Induced Inhibition on the Insulin-Stimulated Akt Phosphorylation

**DOI:** 10.1371/journal.pone.0035783

**Published:** 2012-04-20

**Authors:** Yudong Liu, Ying Su, Shenggang Sun, Tao Wang, Xian Qiao, Xiaoqin Run, Zhihou Liang

**Affiliations:** 1 Department of Orthopedics, Union Hospital, Tongji Medical College, Huazhong University of Science and Technology, Wuhan, Hubei, China; 2 Department of Neurology, Union Hospital, Tongji Medical College, Huazhong University of Science and Technology, Wuhan, Hubei, China; 3 Department of General Surgery, Union Hospital, Tongji Medical College, Huazhong University of Science and Technology, Wuhan, Hubei, China; Virgen Macarena University Hospital, Spain

## Abstract

Evidence has suggested that insulin resistance (IR) or high levels of glucocorticoids (GCs) may be linked with the pathogenesis and/or progression of Alzheimer's disease (AD). Although studies have shown that a high level of GCs results in IR, little is known about the molecular details that link GCs and IR in the context of AD. Abnormal phosphorylation of tau and activation of μ-calpain are two key events in the pathology of AD. Importantly, these two events are also related with GCs and IR. We therefore speculate that tau phosphorylation and μ-calpain activation may mediate the GCs-induced IR. Akt phosphorylation at Ser-473 (pAkt) is commonly used as a marker for assessing IR. We employed two cell lines, wild-type HEK293 cells and HEK293 cells stably expressing the longest human tau isoform (tau-441; HEK293/tau441 cells). We examined whether DEX, a synthetic GCs, induces tau phosphorylation and μ-calpain activation. If so, we examined whether the DEX-induced tau phosphorylation and μ-calpain activation mediate the DEX-induced inhibition on the insulin-stimulated Akt phosphorylation. The results showed that DEX increased tau phosphorylation and induced tau-mediated μ-calpain activation. Furthermore, pre-treatment with LiCl prevented the effects of DEX on tau phosphorylation and μ-calpain activation. Finally, both LiCl pre-treatment and calpain inhibition prevented the DEX-induced inhibition on the insulin-stimulated Akt phosphorylation. In conclusion, our study suggests that the tau phosphorylation and μ-calpain activation mediate the DEX-induced inhibition on the insulin-stimulated Akt phosphorylation.

## Introduction

Insulin resistance (IR) is defined as a state in which normal amounts of insulin produce a subnormal biological response [1]. Binding of insulin to the insulin receptor promotes Akt phosphorylation (and activation) at Ser-473 (pAkt) [2]. Inhibition on the insulin-stimulated Akt phosphorylation is commonly used as a marker of IR [2]. IR is a feature of type 2 diabetes mellitus (DM2) and is involved in a wide spectrum of other clinical diseases. It is increasingly recognized that IR may be linked with Alzheimer's disease (AD) [3], [4]. For instance, clinical investigations suggest that IR may occur before the onset of mild cognitive impairment (MCI) and may be a marker of AD risk [5], [6]. Moreover, rodent studies show that IR increases tau phosphorylation and β-amyloid production, two hallmarks of AD pathology [7], [8]. Furthermore, IR exacerbates memory deficits in transgenic mouse models of AD [9], [10]. Hence, these studies have led to the qualification of AD as ‘an insulin-resistant brain state’. However, little is known about the pathogenic mechanisms that impair insulin action in the context of AD.

Glucocorticoids (GCs) are well known to induce IR in a variety of clinical and experimental settings [11]. In chronic IR and DM2, the concentration of circulating GCs increases. AD patients may also have higher plasma GCs levels than normal controls [12]. A high level of GCs has been shown to result in impaired synaptic plasticity, decreased neurogenesis and hippocampal atrophy, thereby yielding memory deficits [13], [14]. It is thus suggested that there may be a link between a high level of GCs and AD pathogenesis [12], [13], [14]. Although a high level of GCs is a confounding factor in both IR and AD, little is known about the molecular link between GCs and IR in the context of AD.

Tau hyperphosphorylation is one of the hallmark pathological alterations in AD [15]. Tau is a microtubule-stabilizing protein and supports the microtubule system responsible for intracellular transport, axonal morphology and cell physiology. Tau hyperphosphorylation occurs long before the onset of AD symptoms and may play a crucial role in neurodegeneration in AD [15], [16]. Although it has been suggested that tau hyperphosphorylation may contribute independently to AD pathogenesis and/or progression [17], [18], little is known about the molecular mechanisms underlying tau-mediated toxicities. Because both GCs and IR have been shown to lead to tau hyperphosphorylation [19], [20], it is reasonable to postulate that GCs and IR may be linked by tau. We have previously shown that tau might mediate the GCs-induced regulation of cAMP-dependent kinase and cAMP response element binding protein in HEK293 cells stably expressing the longest human tau isoform (tau-441; HEK293/tau441 cells) [21]. We were interested to examine whether tau mediates the GCs-induced IR.

Calpains, a family of calcium-activated cysteine proteases, have been reported to be involved in the pathogenesis of AD [22], [23]. Our previous work and other studies have shown that μ-calpain (also called calpain 1) of the calpains family may play a significant role in AD [24], [25]. Dysregulation of calpains is also associated with the risk of IR and DM2 [26], [27], [28], [29]. Although many studies have focused on calpain 10, more recent evidence also highlights the role of μ-calpain in IR and DM2 [30], [31]. Moreover, activation of μ-calpain is also implicated in the GCs-induced toxicities in muscles and brains [32], [33]. We thus assumed that GCs and IR may be also linked by μ-calpain. It has been shown that activation of μ-calpain mediates the tau-induced toxicities in rats cortical neurons over-expressing human tau [34]. We thus wanted to examine whether μ-calpain also mediates the GCs-induced IR, and if so, whether μ-calpain is linked with tau.

To address the issues mentioned above, we employed two cell lines, wild-type HEK293 cells HEK293/tau441 cells, a good cellular model to study tau pathology [35], [36]. We examined whether DEX, a synthetic GCs, inhibits the insulin-stimulated Akt phosphorylation and if so, whether tau or μ-calpain is involved in the inhibitory effect of DEX. In HEK293/tau441 cells, we made the following observations. 1) DEX increases tau phosphorylation and induces tau-mediated μ-calpain activation. 2) The effects of DEX on tau phosphorylation and μ-calpain activation are prevented by LiCl pre-treatment. 3) Either LiCl pre-treatment or calpain inhibition prevents the DEX-induced inhibition on the insulin-stimulated Akt phosphorylation.

## Materials and Methods

### 1. Antibodies and chemicals

Mouse monoclonal antibody (mAb) against β-actin was purchased from Santa Cruz Biotechnology (Santa Cruz, CA, USA). Rabbit mAb against Akt and phosphorylated Akt at Ser473 (pAkt) were obtained from Cell Signaling Technology (Beverly, MA, USA). Mouse mAb against μ-calpain was purchased from Sigma (St. Louis, MO, USA). Mouse mAb against Tau-1 was obtained from Millipore (Billerica, MA, USA). The antibody R134d that recognizes total tau was generously provided by Gong Cheng-Xin (NYS Institute for Basic Research, SI, NY, USA). Dexamethasone (DEX), insulin, mifepristone (RU), lithium chloride (LiCl) and E-64d (calpain inhibitor) were purchased from Sigma (St. Louis, MO, USA).

### 2. Cell lines, culture and treatment

HEK293 cells stably transfected with the longest isoform of recombinant human tau (tau441; HEK293/tau441) and HEK293 cells transfected with an empty vector (HEK293/vec) were a generous gift from Professor Qing Tian (Department of Pathophysiology, Tongji Medical College, Huazhong University of Science and Technology). The construction of HEK293/tau441 and HEK293/vec cells is as previously described [35], [36]. For control purposes, wild-type HEK293 cells were used and compared with HEK293/vec cells; no differences between the two cell lines were found. Both wild-type HEK293 and HEK293/tau441 were grown in Dulbecco's modified Eagle's medium (DMEM; Invitrogen, Eggenstein, Germany), supplemented with 10% (v/v) fetal bovine serum (FBS; Gibco, Carlsbad, CA, USA) and maintained in 37°C and 5% CO2.

For the experiments, both HEK293/tau441 and wild-type HEK293 cells were first incubated in DMEM with 2% FBS overnight in order to reduce the serum effects. The cells were then pre-treated with RU (20 µM, 30 minutes), LiCl (10 mM, 1 hour), or E-64d (30 µg/ml, 1 hour) and then treated with DEX (1 µM), or treated with DEX (1 µM) alone. For treatment longer than three days, the medium was changed at 72 hours (h) after exposure to DEX. Then RU (20 µM), LiCl (10 mM) or E-64d (30 µg/ml) with DEX (1 µM) or DEX (1 µM) were added to the media. After incubation with the above agents, cells were exposed to 100 nM insulin for 15 minutes (min) and then harvested for Western blotting.

### 3. Western blotting

The total proteins from the cultures were extracted with the ice-cold buffer containing 50 mM Tris-HCl (pH 7.4), 150 mM NaCl, 0.5% sodium deoxycholate, 1% NP-40, 0.1% SDS, 100 µM sodium orthovanadate, 1 mM phenylmethysulfonyl fluoride and 1 µg/ml protease inhibitor cocktail (Beyotime Biotechnology, Haimen, Jiangsu, China). The cell lysates were boiled, briefly sonicated and centrifuged at 12500×*g* for 15 min. The protein concentration in each lysate was determined using a bicinochonic acid (BCA) Protein Assay Kit (Beyotime Biotechnology, Haimen, China) with the bovine serum albumin as the standard. Equal amounts of proteins (60–80 µg) were separated on 10% SDS-polyacrylamide gel (SDS-PAGE) and transferred to nitrocellulose membranes. The membranes were blocked in 5% nonfat milk in TTBS (10 mM Tris-HCl, 150 mM NaCl, 0.02% Tween-20, pH 7.5) and probed overnight at 4°C with antibodies against β-actin (1∶400), pAkt, Akt, Tau-1 and μ-calpain (1∶1000) and total tau (R134d, 1∶10 000). The blots were developed with horseradish peroxidase-conjugated secondary anti-mouse (1∶ 1000; Thermo scientific, Rockford, IL, USA) or anti-rabbit antibodies (1∶ 3000; Signalway antibody, Pearland, TX, USA) and visualized with an enhanced chemiluminescent substrate kit (Thermo scientific, Rockford, IL, USA) and exposure to CL-XPosure film (Kodak). The films were scanned and the density of each of band was quantified using Kodak Digital Science 1D software (Eastman Kodak Co., New Haven, CT).

### 4. Statistical analysis

Numerical data were expressed as group means ± SEM and analyzed with SPSS statistical software (version 13.0). All data sets were subjected to one-way ANOVA before application of *post hoc* pair-wise comparisons. *P*<0.05 or lower was considered to represent statistically significant differences.

## Results

### 1. DEX induces inhibition on the insulin-stimulated Akt phosphorylation in wild-type HEK293 and HEK293/tau441 cells

**Figure 1:**
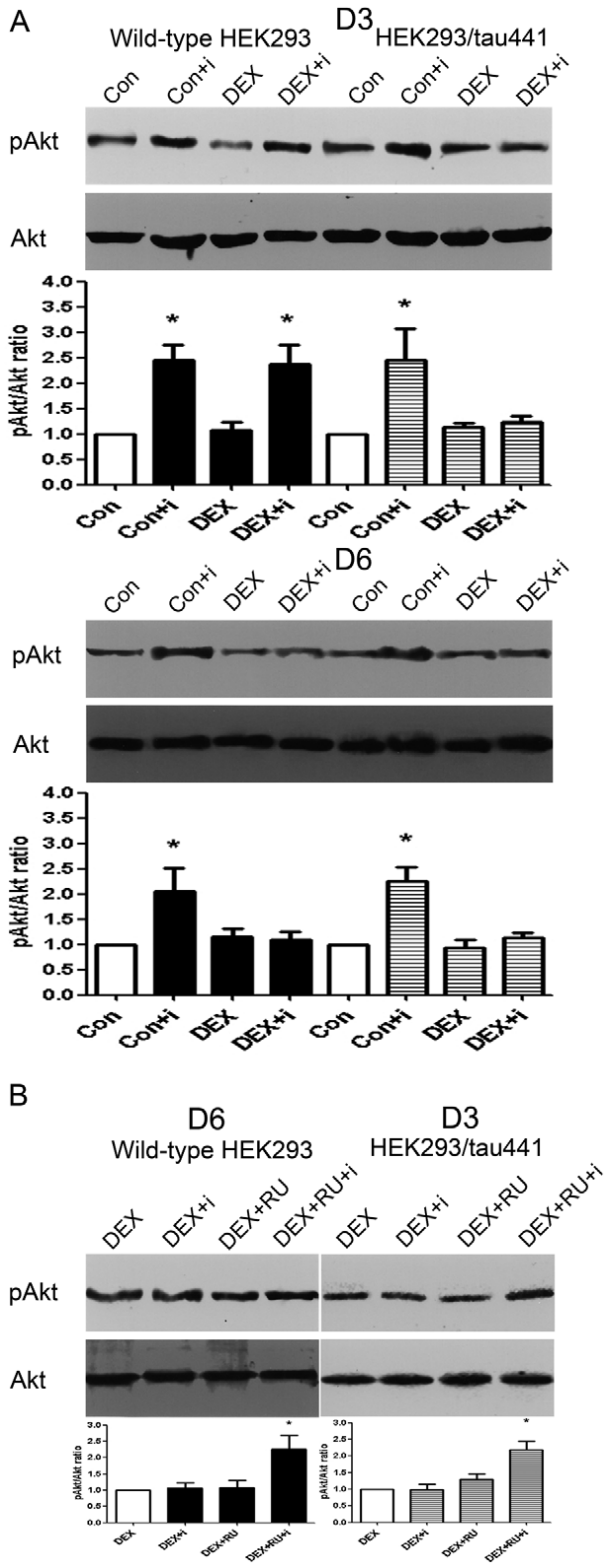
Effects of dexamethasone on the insulin-stimulated Akt phosphorylation. A): Both wild-type HEK293 and HEK293/tau441 cells were treated with 1 µM dexamethasone (DEX) for 1–6 days and then stimulated with 100 nM insulin (i) for 15 min. Representative immunoblots from the results on the third day (D3) and the sixth day (D6) after DEX treatment were shown. Bars representing means ± SEM were shown below. Total amounts of Akt remained stable under the conditions. In HEK293/tau441 cells, DEX prevented the insulin-stimulated increases in pAkt on D3 and D6. In wild-type HEK293 cells, the inhibitory effect of DEX was evident on D6 but not on D3. Each experiment was repeated three times unless stated otherwise. ^*^ *P*<0.05 versus control. B): Culture cells were pre-treated with mifepristone (RU; 20 µM, 30 min) and then treated with DEX for 3 days or 6 days. Representative immunoblots from wild-type HEK293 cells on D6 and those from HEK293/tau441 cells on D3 were shown, with bars representing means ± SEM below. Pre-treatment with RU prevented the inhibitory effects of DEX. ^*^ *P*<0.05 versus DEX group.

The insulin-stimulated increase in pAkt is a key event in the proximal insulin signalling pathway and the pAkt/Akt ratio has been widely used as a marker for evaluating IR [2]. We therefore determined whether GCs induce IR by examining the insulin-stimulated Akt phosphorylation (pAkt/Akt ratio) in wild-type HEK293 and HEK293/tau441 cells. Dexamethasone (DEX), a synthetic GCs and specific glucocorticoid receptor (GR) agonist, was used in our study as GCs. The concentration of DEX was 1 µM because it is in the range of the amount given for the therapeutic purpose in clinical practice (e.g., dexamethasone suppression test, prevention of respiratory distress in preterm infants) and also applied for induction of long-term stress [37], [38]. Long-term (1–6 days) treatment with DEX was applied to both cell lines (see *Materials and Methods*) to mimic the induction of long-term stress *in vivo* [37], [39]. Representative immunoblots from the results obtained on the third day (D3) and on the sixth day (D6) after DEX treatment were shown in Fig. 1. As shown in Fig. 1, total amounts of Akt remained stable under the conditions and were used as internal standards for measurement of Akt phosphorylation. Under basal conditions, insulin induced similar increases in pAkt in both cell lines (lane 2 and 6, Fig. 1A). This suggests that in HEK293/tau441 cells, the exogenously expressed tau by itself does not affect the insulin-stimulated Akt phosphorylation. Treatment with DEX alone did not affect the basal pAkt levels in either of the two cell lines (lane 3 and 7, Fig. 1A). In wild-type HEK293 cells, the inhibitory effect of DEX was not evident until D6 (D6, lane 4, Fig. 1A). In HEK293/tau441 cells, the inhibitory effect of DEX occurred on D3 (D3, lane 8, Fig. 1A) and then sustained on day 4, day 5 (data not shown) and D6 (D6, lane 8, Fig. 1A). We thus chose D3 and D6 as two representative time points for the following experiments.

There two types of receptors that GCs can bind with, one is glucocorticoid receptor (GR) and the other is mineralcorticoid receptor (MR). Because HEK293 cells do not express MR [40], we only used mifepristone (RU), a GR antagonist. Representative immunoblots from wild-type HEK293 cells on D6 and those from HEK293/tau441 cells on D3 were shown in Fig. 1B. Without insulin stimulation, pre-treatment with RU (20 µM, 30 min; see *Materials and Methods*) did not affect the pAkt levels upon DEX treatment (lane 3, Fig. 1B). However, pre-treatment with RU prevented the inhibitory effects of DEX in both cell lines (lane 4, Fig. 1B), indicating that the inhibitory effect of DEX is mediated by the GR.

Although DEX inhibited the insulin-stimulated Akt phosphorylation in wild-type HEK293 and HEK293/tau441 cells, the inhibitory effect of DEX occurred earlier in HEK293/tau441 cells (D3 *versus* D6). Because GCs are known to induce tau phosphorylation [14], we speculate that it is the DEX-induced increase in tau phosphorylation that causes the difference described above.

### 2. DEX increases tau phosphorylation in HEK293/tau441 cells, which is prevented by lithium chloride

**Figure 2:**
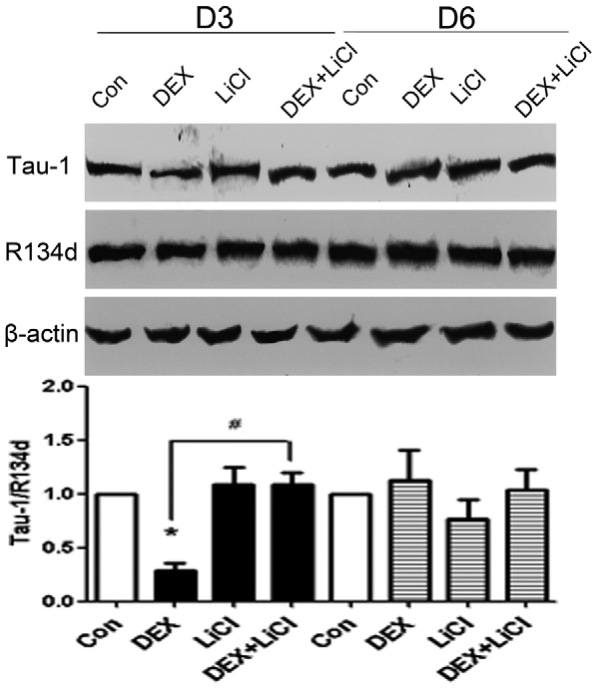
Effects of DEX and lithium chloride on tau phosphorylation. HEK293/tau441 cells were pre-treated with lithium chloride (LiCl; 10 mM, 1 h) and then treated with DEX for 3 days (D3) or 6 days (D6), followed by Western blotting analysis of tau phosphorylation with Tau-1 (against dephosphorylated tau within the epitope 189–207) and R134d (against total tau) antibodies. Total amounts of tau remained largely stable and the level of tau phosphorylation was determined by the Tau-1/R134d ratio. Bars representing means ± SEM. On D3, DEX induced an increase in tau phosphorylation, as evidenced by the decrease in the Tau-1/R134d ratio. Pre-treatment with LiCl prevented the effect of DEX on tau phosphorylation on D3. ^*^ *P*<0.05 versus control, ^#^ *P*<0.05 between indicated groups.

We next assessed whether DEX increases tau phosphorylation in HEK293/tau441 cells, and if so, whether lithium choride (LiCl), an inhibitor of glycogen synthase kinase-3β (GSK-3β) that has been shown to inhibit tau hyperphosphorylation in different contexts [41], [42], [43], can prevent the action of DEX. To this end, Western blots were performed using Tau-1 and R134d antibodies. Tau-1 is an antibody that recognizes tau when it is dephosphorylated within the epitope 189–207 [44]. R134d is an antibody that recognizes total tau. HEK293/tau441 cells were pre-treated with LiCl (10 mM, 1 h) and then treated with DEX (see *Materials and Methods*) for 3 days (D3) or 6 days (D6). Total amounts of tau (detected by R134d) remained stable under the conditions and were used as internal standards for measurement of the relative level of Tau-1 (Tau-1/R134d ratio, Fig. 2). As expected, DEX induced an increase in tau phosphorylation on D3, as evidenced by the decrease in the level of Tau-1 (lane 2, Fig. 2). However, DEX did not affect the Tau-1 level on D6 (lane 6, Fig. 2). LiCl treatment alone did not affect basal Tau-1 levels (lane 3 and 7, Fig. 2). However, pre-treatment with LiCl prevented the DEX-induced decrease in Tau-1 on D3 (lane 4, Fig. 2), suggesting that the DEX-induced increase in tau phosphorylation is prevented by LiCl. Interestingly, although LiCl is widely accepted as an inhibitor to GSK-3β [41], GSK-3β activity, measured by the ratio of the phosphorylated GSK-3β at Ser-9 (p GSK-3β) and total GSK-3β, was not obviously affected by DEX in HEK293/tau441 cells on D3 (data not shown). DEX actually decreased GSK-3β activity (an increase in pGSK-3β/GSK-3β ratio) in wild-type HEK293 cells on D3 (data not shown). The mechanisms responsible for the effects of DEX and LiCl on tau phosphorylation in our system warrants further research. Nevertheless, results described above show that LiCl pre-treatment prevents the DEX-induced increase in tau phosphorylation in HEK293/tau441 cells.

### 3. Insulin does affect tau phosphorylation in HEK293/tau441 cells

**Figure 3:**
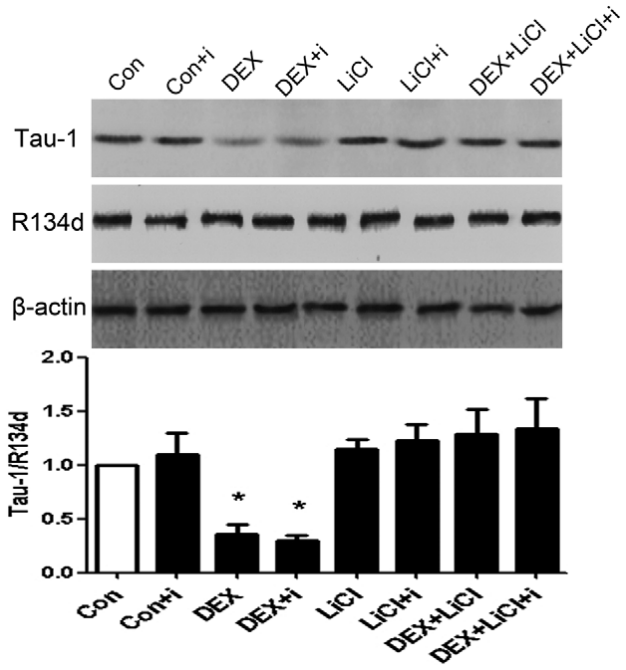
Effects of insulin on tau phosphorylation. HEK293/tau441 cells were pre-treated with LiCl (10 mM, 1 h) and then treated with DEX for 3 days, followed by stimulation of insulin for 15 min and Western analysis of Tau-1 and R134d. Bars representing means ± SEM. Insulin did not prevent the DEX-induced decrease in Tau-1 or affected Tau-1 levels under other conditions, suggesting that insulin does not affect tau phosphorylation. ^*^ *P*<0.05 versus control.

We have shown that LiCl prevented the DEX-induced increase in tau phosphorylation in HEK293/tau441 cells. To test our hypothesis that the DEX-induced increase in tau phosphorylation mediates the inhibition on the insulin-stimulated Akt phosphorylation, we should examine whether LiCl corrects the inhibitory effects of DEX. However, before that we also wanted to see how insulin affects tau phosphorylation because previous studies demonstrated that insulin could induce either tau phosphorylation or dephosphorylation [45], [46], [47]. Moreover, insulin is well known to activate the Akt/GSK-3β signaling pathway and thus inhibits the activity of GSK-3β [45], [46]. Because we have shown GSK-3β inhibition by LiCl prevented the DEX-induced tau phosphorylation (Fig. 2), we examined the effect of insulin on tau phosphorylation. HEK293/tau441 cells were pre-treated with LiCl (10 mM, 1 h) and then treated with DEX (see *Materials and Methods*) for 3 days (D3), followed by insulin stimulation. Total amounts of tau remained stable under the conditions (Fig. 3). Insulin stimulation under basal condition did affect the Tau-1 level (lane 2, Fig. 3). Upon treatment with LiCl or co-treatment with DEX and LiCl, insulin did not affect Tau-1 levels either (lane 6 and 8, Fig. 3). Importantly, insulin did not prevent the DEX-induced decrease in Tau-1 (lane 4, Fig. 3). These results suggest that insulin stimulation, at least in our system, does not affect tau phosphorylation.

### 4. Lithium chloride prevents the DEX-induced inhibition on the insulin-stimulated Akt phosphorylation in HEK293/tau441

**Figure 4:**
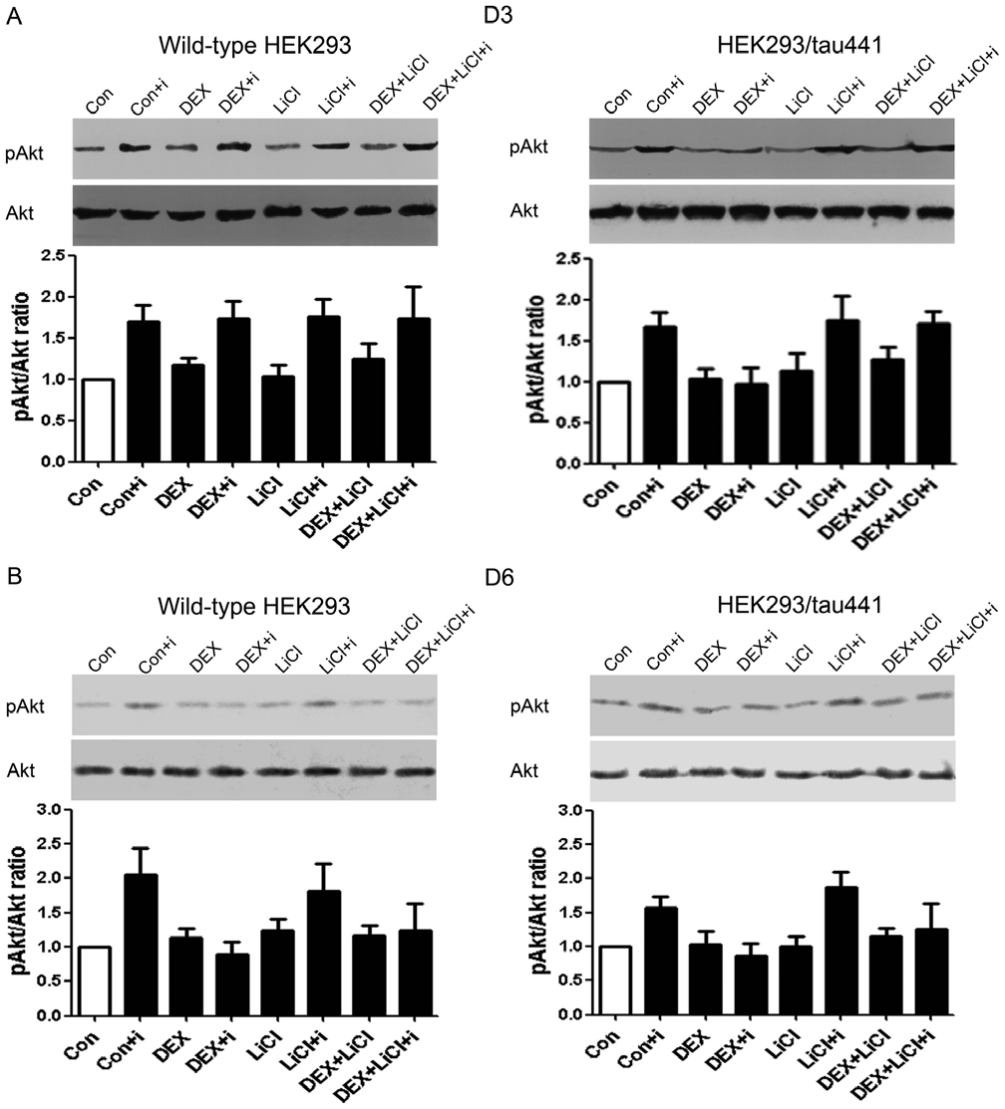
Effects of LiCl on the DEX-induced inhibitory effect. Both wild-type HEK293 and HEK293/tau441 cells were pre-treated with LiCl (10 mM, 1 h) and then treated with DEX for 3 days (D3; A) or 6 days (D6; B), followed by stimulation of insulin and Western analysis of pAkt and Akt. Bars representing means ± SEM. Pre-treatment with LiCl prevented the inhibitory effect of DEX in HEK293/tau441 cells on D3.

We have shown that LiCl prevented the DEX-induced increase in tau phosphorylation and insulin did not affect tau phosphorylation. We next examined whether LiCl corrects the DEX-induced inhibition on the insulin-stimulated Akt phosphorylation in HEK293/tau441 cells. However, it has also been reported that LiCl affected insulin signaling through tau-independent mechanisms [48]. We therefore also examined how LiCl affects the insulin-stimulated Akt phosphorylation in wild-type HEK293 cells. Both cell lines were treated with LiCl, DEX, or in combination of them for 3 days (D3) or 6 days (D6). In both cell lines, LiCl did not have obvious effects on pAkt levels under basal conditions, under insulin stimulation alone or upon DEX treatment alone (lane 5, 6 and 7, Fig. 4). LiCl prevented the inhibitory effect of DEX in HEK293/tau441 cells on D3 (HEK293/tau441, lane 8, Fig. 4A). However, on D6, when the inhibitory effects of DEX were evident in both cell lines (lane 4, Fig. 4B), pre-treatment with LiCl did not seem to have obvious effects on the DEX-induced inhibition in either of the cell lines on D6 (lane 8, Fig. 4B). Notice that the DEX-induced increase in tau phosphorylation was evident on D3 but not on D6 in HEK293/tau441 cells (Fig. 2). Taken together, the above results suggest that the DEX-induced increase in tau phosphorylation may mediate the inhibitory effect of DEX in HEK293/tau441 cells.

### 5. DEX induces tau-mediated μ-calpain activation and inhibition of calpain activity prevents the negative effects of DEX in HEK293/tau441 cells

**Figure 5:**
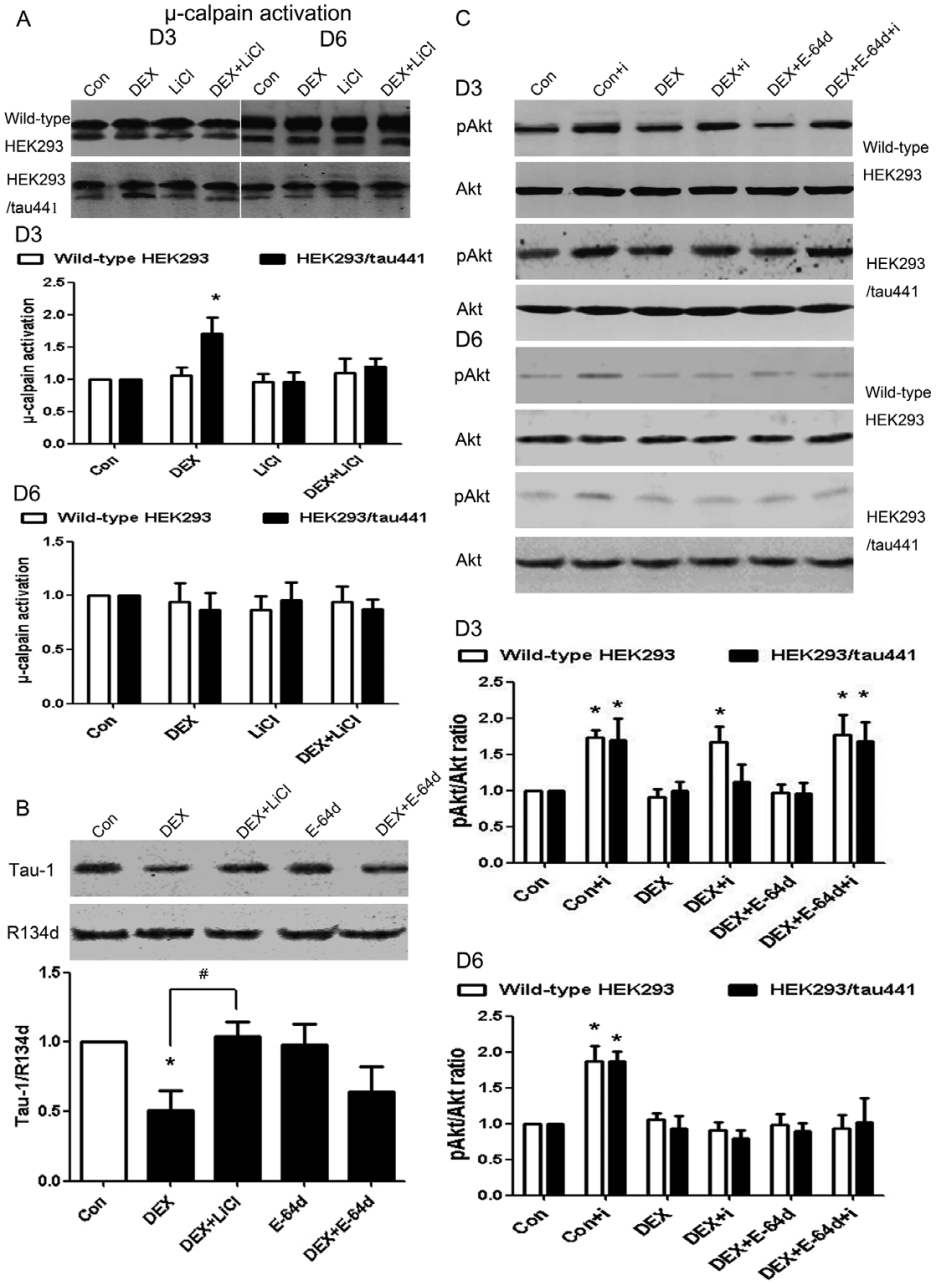
Involvement of μ-calpain in the inhibitory effect of DEX. A): Both wild-type HEK293 and HEK293/tau441 cells were pre-treated with LiCl (10 mM, 1 h) and then treated with DEX for 3 days (D3) or 6 days (D6). Activation of μ-calpain was determined by the ratios of the active/truncated calpain (78-kDa bands) and the inactive/full-length calpain (80-kDa bands). Bars representing means ± SEM. On D3 in HEK293/tau441 cells, DEX induced μ-calpain activation and pre-treatment with LiCl prevented the activation of μ-calpain. ^*^ *P*<0.05 versus control. B): HEK293/tau441 cells were pre-treated with E-64d (30 µg/ml, 1 h) or LiCl (10 mM, 1 h), and then treated with DEX for 3 days. The level of tau phosphorylation was determined by the ratio of Tau-1/R134d. Bars representing means ± SEM. E-64d did not have obvious effect on the DEX-induced increase in tau phosphorylation. ^*^ *P*<0.05 versus control, ^#^ *P*<0.05 between indicated groups. C) Both wild-type HEK293 and HEK293/tau441 cells were pre-treated with E-64d (30 µg/ml, 1 h) and then treated with DEX for 3 days (D3) or 6 days (D6), followed by stimulation of insulin and Western analysis of pAkt and Akt. Bars representing means ± SEM. Pre-treatment with E-64d prevented the inhibitory effect of DEX in HEK293/tau441 cells on D3. ^*^ *P*<0.05 versus control.

We have shown that the DEX-induced increase in tau phosphorylation mediated the inhibitory effect of DEX in HEK293/tau441 cells. According to our hypothesis, we further determined if μ-calpain activation also mediates the inhibitory effects of DEX, and if so, whether the DEX-induced μ-calpain activation is mediated by tau phosphorylation. Activation of μ-calpain was determined by the ratio of the active/truncated calpain (78-kDa band) and the inactive/full-length calpain (80-kDa band). DEX did not affect μ-calpain activation in wild-type HEK293 cells on D3 or on D6 (lane 2, Fig. 5A). DEX did not affect μ-calpain activation in HEK293/tau441 cells on D6 either (D6, lane 6, Fig. 5A). In contrast, DEX induced an increase in μ-calpain activation in HEK293/tau441 cells on D3 (D3, lane 2, Fig. 5A). Because the DEX-induced increase in tau phosphorylation was evident on D3 (lane 2, Fig. 2), the above results suggest that the DEX-induced tau phosphorylation may correlate with μ-calpain activation in HEK293/tau441 on D3. However, it has been shown that μ-calpain could result in tau phosphorylation [49]. To examine whether μ-calpain activation mediates the DEX-induced tau phosphorylation, we pre-treated HEK293/tau441 cells with E-64d (30 µg/ml, 1 h), a cell-membrane-permeable calpain inhibitor, and then determined the levels of Tau-1 and total tau by Western blotting. As Fig. 5B shows, pretreatment with E-64d did not prevent the DEX-induced decrease in Tau-1 in HEK293/tau441 cells. This suggests that μ-calpain may not mediate the DEX-induced tau phosphorylation. In contrast, pre-treatment with LiCl (10 mM, 1 h) prevented the DEX-induced μ-calpain activation in HEK293/tau441 cells on D3 (D3, lane 4, Fig. 5A). Notice that LiCl did not affect μ-calpain activation in HEK293/tau441 cells on D6 or in wild-type HEK293 cells (Fig. 5A). Because μ-calpain activation was observed only in the tau-expressing HEK293/tau441 cells and prevention of tau phosphorylation by LiCl inhibited the DEX-induced μ-calpain activation, these results suggest that the DEX-induced tau phosphorylation mediates μ-calpain activation in HEK293/tau441 cells.

Next we assessed whether the DEX-induced and tau-mediated μ-calpain activation mediates the DEX-induced inhibition on the insulin-stimulated Akt phosphorylation on D3. However, previous studies have shown that calpain may inhibit insulin signaling [50]–[53]. We also examined how calpain inhibition would affect the inhibitory effect of DEX on D6, when no activation of μ-calpain was observed upon DEX treatment (D6, lane 2, Fig. 5A). Both cell lines were pre-treated with E-64d (30 µg/ml, 1 h) and then treated with DEX for 3 days (D3) or 6 days (D6) (See *Materials and Methods*). In wild-type HEK293 cells, E-64d did not affect the insulin-stimulated increase in pAkt upon DEX treatment on D3 (wild-type HEK293, D3, lane 6, Fig. 5C) or prevented the inhibitory effect of DEX on D6 (wild-type HEK293, D6, lane 8, Fig. 5C). In HEK293/tau441 cells, E-64d did not prevent the inhibitory effect of DEX on D6 (HEK293/tau441, D6, lane 6, Fig. 5C). Notice the DEX-induced tau phosphorylation was not evident on D6 (Fig. 2). On D3, when the DEX-induced tau phosphorylation was evident (Fig. 2), E-64d prevented the inhibitory effect of DEX (HEK293/tau441, D3, lane 6, Fig. 5C). These results suggest that the involvement of μ-calpain in the inhibitory effect of DEX may depend on tau phosphorylation in our system. Taken together, the above results suggest that the DEX-induced μ-calpain activation may also mediate the inhibitory effect of DEX. Moreover, the involvement of μ-calpain seems to depend on the DEX-induced tau phosphorylation.

## Discussion

In the current study, several observations have been made using a cellular model system for studying tau pathology, i.e., a HEK293 cell line stably expressing the longest isoform of human tau (HEK293/tau441 cells). First, DEX increased tau phosphorylation and induced tau-mediated μ-calpain activation. Second, the effects of DEX on tau phosphorylation and μ-calpain activation were prevented by LiCl pre-treatment. Third, both LiCl pre-treatment and calpain inhibition by E-64d prevented the DEX-induced inhibition on the insulin-stimulated Akt phosphorylation.

Numerous potential mechanisms have been proposed to explain the pathogenic mechanisms of IR [54]. However, these studies mainly focused in three main targets, i.e., liver, skeletal muscle and adipose tissue [55], [56]. The origin of IR in the context of AD is difficult to elucidate due to the diverse set of risk factors linked to AD. Our results suggest that the GCs-induced tau phosphorylation and consequent μ-calpain activation may mediate IR. This suggests that GCs and IR, two potent driving forces for AD [4], [13], may be linked by tau phosphorylation in the context of AD. However, neither LiCl nor E-64d prevented the inhibitory effect of DEX on D6, when the inhibitory effects of DEX were evident in both cell lines. This suggests that the involvement of the GCs-induced tau phosphorylation and consequent μ-calpain activation in IR may be only within a particular time window.

Previous rodent studies suggest that IR or lack of insulin signaling can result in tau hyperphosphorylation [20], [57], [58]. Indeed, it is generally accepted that insulin activates Akt signaling, inhibits GSK-3β activity and thus may reduce tau phosphorylation [45]. It is reasonable to speculate that IR may lead to tau phosphorylation by activating GSK-3β. However, our results show that insulin does not affect tau phosphorylation under basal condition or upon treatment with DEX (Fig. 3). These results suggest that i) insulin signaling may not affect tau phosphorylation, and ii) the DEX-induced tau phosphorylation is not mediated by inhibition of the insulin signaling in our system. In fact, there is a debate about the relationship with the insulin-activated Akt/GSK-3β signaling and tau phosphorylation. In brain/neuron-specific insulin receptor knockout mice, lack of insulin signaling in the brain activates GSK-3β and leads to tau hyperphosphorylation [57]. Intriguingly, in high fat diet C57BL6 mice, IR actually reduces tau phosphorylation at Ser396, Ser202/Thr205 and Thr231 [59]. Moreover, in C57BL mice, insulin administration does not affect tau phosphorylation at GSK-3β-relevant sites but promotes tau phosphorylation at sites that are dependent on extracelluar regulated kinase [60]. Interestingly, insulin transiently promotes tau phosphorylation by activating GSK-3β in SH-SY5Y neuroblastoma cells and primary cortical neurons [46], [61]. Furthermore, in C57BL mice intraperitoneally injected with streptozotocin, which leads to insulin depletion, tau phosphorylation is mediated by the decrease in protein phosphatase 2A activity but not by the increase in GSK-3β activity [58]. How lack of insulin signaling and IR affect tau phosphorylation *in vivo* and in AD patients warrants further research. Nevertheless, our study suggests that i) the GCs-induced tau phosphorylation may not be mediated by inhibition of insulin signaling, and ii) the GCs-induced tau phosphorylation could function upstream as a mediator of IR.

Clinical observations suggest that DM2 is among the risk factors for AD [5], [6], [62]–[64]. Because tau hyperphosphorylation is one of the key pathological features of AD, our results that the DEX-induced tau phosphorylation could occur upstream to IR do not seem to be supported by these clinical data. However, it should be noticed that tau hyperphosphorylation and the detectable clinical symptoms of AD are two different notions. Tau hyperphosphorylation may actually occur decades before individuals manifest clinical symptoms of AD [65]. Tau phosphorylation may also increase during normal aging [66]. Thus it is not surprising that tau hyperphosphorylation also occurs in asymptomatic patients with Braak-stage I neuropathology where clinical symptoms of AD are absent [65]. Because tau hyperphosphorylation can occur long before the onset of symptoms and may play a key role in AD pathogenesis and/or progression, it is possible that tau hyperphosphorylation could contribute to the brain IR in AD. Because both tau phosphorylation and the prevalence of IR increases with aging [67], it might be difficult to identify which of these two events first occurs in brain. It should be also noticed that brain IR and peripheral IR may be two different pathological alterations [68]. The extant literature does not resolve whether the brain IR in AD represents a local disease process, or complication/extension of peripheral IR, i.e. chronic hyperglycemia, hyperinsulinemia, and DM2.

Although the inhibitory effects of DEX were evident in both cell lines on D6, calpain inhibition did not prevent the inhibitory effects in either of the cell lines (Fig. 5C). In contrast, calpain inhibition prevented the inhibitory effect of DEX only on D3, when the DEX-induced tau phosphorylation was evident (Fig. 2 and Fig. 5C). These results suggest that the involvement of μ-calpain in the GCs-induced IR may not be a universal phenomenom and the action of μ-calpain seems to depend on the GCs-induced tau phosphorylation. The roles of calpains in the pathogenesis of IR and DM2 have been extensively studied [28], [29], [30], [31]. Although Calpain 10 is the first identified diabetes gene through a genome scan [69]., little is known about its protease activity and physiological functions [70]. Furthermore, whether calpain 10 really links with IR and DM2 is still in controversy [70]–[72]. Some recent studies have suggested that μ-calpain activation may contribute to the inhibition of insulin signaling. For instance, μ-calpain cleaves phosphoinositide 3-kinases (PI3Ks), an important upstream activator of Akt, and reduces PI3Ks enzymatic activities in NIH 3T3 cells [53]. Moreover, μ-calpain degrades insulin receptor substrates (IRS), major substrates of insulin signaling, in human neuroblastoma cells and prostate epithelial cells [50], [52]. Furthermore, calpain activation by calcium incubation reduced total amount of Akt and heat shock protein 90, a key Akt regulator protein, in rats skeletal muscle cells [51]. Although these studies suggest that μ-calpain activation may mediate IR, other studies also show contradictory results. For instance, calpain inhibition by over-expression of calpastatin (the endogenous calpain inhibitor) induced a decrease in Akt level and subsequent IR in mice [73]. Moreover, in soleus muscle strips and adipocytes and adipocytes isolated from Wistar rats, calpain inhibition by E-64d reduced insulin-mediated signaling [74]. Furthermore, in 3T3-L1 adipocytes and HepG2 hepatoma cells, inhibition of calpain did not affect the insulin-mediated PI3K/Akt pathway but it did prevent the insulin-stimulated glucose uptake [75], [76]. These studies suggest that the roles of μ-calpain in IR may be tissue-specific. Our results suggest that the involvement of μ-calpain in IR, at least in tau-expressing tissues, may depend on the GCs-induced tau phosphorylation.

However, how the GCs-induced tau phosphorylation mediates μ-calpain activation is not known. The activation and regulation of calpain activity in vivo is not well understood, although it is a ubiquitously expressed protein with limited proteolytic activity and therefore it is assumed to be tightly regulated [77]. We found an enhanced co-localization of phosphorylated tau and μ-calpain in HEK293/tau441 and SH-SY5Y cells upon DEX treatment (unpublished data). We speculate that the GCs-induced tau phosphorylation may enhance the structural link between tau and μ-calpain and thus mediate the activation of μ-calpain. Interestingly, in a postmortem study of AD patients' brains, it was found that the hyperphosphorylated tau can bind with a calcium-binding protein—EF hand domain protein 2, which may be involved in modification of calpain activity [78].

In conclusion, we have demonstrated that the GCs-induced tau phosphorylation and consequent μ-calpain activation may contribute to IR. Our study suggests that tau phosphorylation and μ-calpain activation may be two key events that link GCs, IR and AD.
